# Digital, big data and computational forensics

**DOI:** 10.1080/20961790.2018.1500078

**Published:** 2018-10-23

**Authors:** Zeno Geradts

**Affiliations:** a Netherlands Forensic Institute, Den Haag, The Netherlands;; b University of Amsterdam, Institute of Informatics, Amsterdam, The Netherlands

Recent years have witnessed significant developments in deep learning and artificial intelligence [1]. For instance, remarkable improvements have been made in automated face comparison systems by using deep learning, compared with the classic approaches [2].

The term “deep learning” is often used to refer to certain kinds of neural networks. The first publications on biological neural networks and the brain date back to the late 1800s [3]. It was not until the rediscovery of the back-propagation algorithm [4] in 1986 that interest in the field was reignited. An artificial neural network is designed following a simple modelling of the brain, and involves a representation of neurons. A neuron receives a specific signal and converts to a different one. Neurons can also be used to learn from examples. They adjust a transfer function. Many neurons are linked together, and are often used in multiple layers. A visual overview is provided in Figure 1.

**Figure 1.**
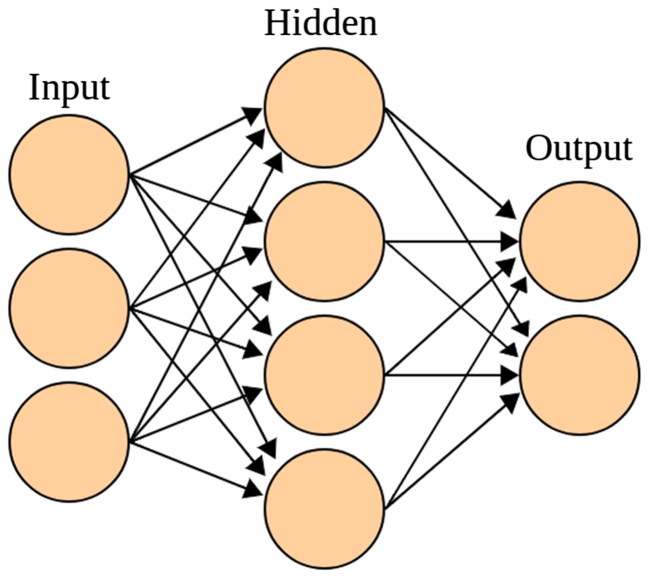
Schematic overview of a neural network (from Wikimedia Commons).

An example of the application of neural networks is face recognition [5], where these networks examine images of people’s faces and find features, such as shapes of nose, ears, and mouth. In such networks, the parameters of thousands or more neurons are adjusted based on training to improve recognition performance. Combined with improved pattern recognition to detect the eyes, mouth, and the position of the face, they yield better results.

This type of network can be used not only for images but also for large amounts of text and other data, including audio. It must be trained for each task on relevant data. In recent years, computation power was been greatly enhanced though Graphical Processing Unites (GPUs) in personal computers (PCs [6]). They have become considerably cheaper over time, and can now be used in parallel.

Neural networks are used for many types of data [7–9]. If the ground truth of a dataset is known, this is good for the training set, since it needs to learn from correct examples. For example, a neural network can be trained to detect with fraudulent as well as benign transactions [10]. This technology can then be applied, and allows for the identification that would not have possible through manual effort.

One problem with neural networks is that they operate like a black box, as the network can be trained on information other than that desired. It is well known that algorithms based on neural networks were used by the US military in the 1980s to recognize enemy artillery tanks hidden in forests [11], and were found to be efficient. In reality, the algorithm did not recognize the tanks, but whether it was sunny or cloudy day, since the training set was made with US tanks in woods during a sunny day and was made with enemy tanks in woods during a cloudy day.

A major advantage of computers is that they can quickly search through large amounts of data. A person becomes tired after manually examining images of faces, and the process is more prone to error [12]. A computer can easily examine several million faces in the same group of facial features. This article discusses definitions of big data relevant to forensics, practical, and ethical considerations of applications and expectations for the future.

## Definition of big data

Many definitions of big data have been offered in the literature [13], and most incorporate the volume, variety, and velocity of the data generated as well as the velocity of the analysis needed.

Other factors are important in studying big data [13]. The quality of data is a concern. Some sources are more reliable than others. Moreover, data can be misinterpreted and false conclusions drawn based on an analysis of trends in big data. Consider the example of the service Google Flu Trends that attempted to predict flu activity. Between 2006 and 2010, it could predict well how the flu developed by examining queries for flu on its search engine. Around 2012, this no longer worked, and so the project was terminated [14].

In forensic science, the term “computational forensics” is also used to refer to the automated analysis of forensic traces, and is related to forensic data science [15].

## Relevance to forensic sciences

In forensics, the automatic fingerprint identification system (AFIS) was introduced in the 1980s [16]. In the early 1990s, using automated systems of bullets and shells made an important step for comparing striation and impression marks. Similar success has been achieved in automating the recognition of marks and scuffs left by tools and shoes [17, 18], with pattern recognition.

Many tools for network-based analysis of data are available for law enforcement. The first ones emerged in the 1990s, but were limited in the amount of data that they could analyze.

Many companies sell software for the automated analysis of digital data secured from such sources as smartphones, computers, and Internet taps. The need to search through these data has led to the development of increasingly advanced search methods.

Seized data from, for example, a smartphone or a computer, are in part structured and partly unstructured. “Structured” in this context means that a part of the data can be interpreted as it is stored in a standard manner, but another part cannot because it is not stored in a standard or known manner, i.e. it is unstructured. Information from emails and social messaging sites are examples of structured data.

The Netherlands Forensic Institute has developed a system for forensic search for the police [19]. It can be used to search through large amounts of data, e.g. digital traces, including emails, instant messages or images, and video material. Location information from photos and other data can also be plotted on a map using this system. It was developed using an open-source big data-handling software, and can be used in as transparent a manner as possible. Furthermore, the system is continually updated to accommodate file formats used by newly developed apps.

Algorithms are available to determine the make and model of the camera using which a given image was taken, and are used in forensic casework [20]. The small artefacts in sensors of cameras between the different light sensitive elements, is of interest in forensic science. The artefacts between pixels make a pattern, the Photo Response Non-Uniformity (PRNU) pattern.

Based on this pattern, a kind of fingerprint of the sensor can be determined, so evidence that an image has been with a specific camera. The algorithms proposed in [21, 22] can be used to identify the camera that was used to make pornographic images of children.

Another example of the use of computer algorithms is the analysis of large amounts of text. Dujin et al. [23] has researched the use of neural networks in criminal databases and arrived at a number of conclusions. They combined various kinds of information concerning relationships among 22 000 known criminals. This made use of data from social media, police reports, and arrest records. These criminals had been convicted of a variety of crimes, such as drug use and trafficking, extortion, money laundering, and manufacturing synthetic drugs.

From this research and simulations, it appears that the common method of law enforcement to arrest the bosses of the criminal organization appears that the organizations become stronger since leaders are easily replaced, whereas for example toxicologist are much harder to replace. The network is a complex system that adapts rapidly to changing circumstances, and it is impossible to obtain a picture of the activities in a given domain in a classical structure.

Grauss et al. [24] examined the relationship between entities in public datasets from digital data related to Enron, including e-mails and social networking traffic. Emerging entities in a network are also important for detecting abnormal behaviour and networking activities between entities of interest to the investigation. They also showed many predictions concerning behaviour can be made using digital data.

These techniques for predictive data analysis may be important for the prevention of crime and predictive policing. There is a growing recognition in criminology of the opportunities offered by big data [25–27]. The increasing number of sensors in technologies used by people, including smartwatches and medical equipment, has enormous potential if data from them are available for analysis. Location information as well as the fitness trackers can serve as evidence in court [28–30].

## Caveats and limitations

In Europe, the General Data Protection Regulation (GDPR) addresses data protection and privacy for all individuals within the European Union (EU). It also addresses the export of personal data outside the EU. Effective in May 2018, the law is expected to have a significant impact on the type and amount of data that can be used within law enforcement, though there are many exceptions. Even in countries with few regulations regarding privacy and ethics, people’s perception of the importance of privacy change as they begin sharing less data about themselves [31–33].

By a clever combination of human effort and the use of computers, a considerable amount of time can be saved. Moreover, companies like Microsoft, Google, and Apple are using this idea in large-scale data analysis for the advertising market [34]. All search queries by users in addition to their location information provides invaluable information to these companies about user preferences. Techniques developed in these areas can be used in forensic research, and many companies have rendered their technologies open source, such as PyTorch, Hadoop, TensorFlow, FaceNet, and OpenFace [35–37].

It is expected that anti-forensic techniques will increase as the privacy-awareness increases. Existing anti-forensic tools can be fully wiped or changed easily [38]. It is also conceivable that wrong tracks are added to datasets to corrupt them [39]. The creation of zombie accounts is an instance [40]. Strong encryption is readily available to users.

Most artificial intelligence-based solutions can only function well for one task at a time [41]. Thus, a good algorithm can recognize faces but can perform no other task. According to some studies, algorithms tend to perform better than humans at such games as chess where computers have been known to beat expert human players [42]. However, they are not as good at judging softer information, such as pictures with weaker associated information. Combining a plurality of such algorithms is an important step. We assume here that these algorithms are used in forensic science as tools and their results are critically verified by human experts.

The widespread use of big data raises several ethical and privacy-related issues [43]. An internal solution for privacy and data protection is in the design of systems [44] as well as policies concerning access.

Predictive policing also uses deep learning. Using training on a sample dataset, the system can predict if crimes will occur in a certain neighbourhood. Discussions are ongoing on the ethical aspects of predictive policing as algorithms might make wrong decisions and discriminate against a group of people [33, 45] with the training sets. Validation and human intervention are thus needed depending on use. A feedback mechanism in the software to learn from bad decisions might help improve it. The system should also provide a level of confidence for its results based on the data.

## The future perspective

Using artificial intelligence in the criminal justice system can accelerate judicial decisions since they can be automated. Naturally, errors in the decisions found using such techniques should also be considered and validated [46, 47].

Predictive policing can ultimately help prevent crime. It is important to consider the social, ethical, legal, and privacy-related issues involved prior to its acceptance. In the coming years, more research is expected on the combination of large biometric databases and surveillance data [48], e.g. big data projects using biometric features of convicted criminals, which also has ethical implications.

Using artificial intelligence and deep learning, court decisions can be predicted [46]. Another interesting research topic is to examine court decisions and locate misinterpretations of (forensic) evidence, which can be useful in tracking wrongful convictions.

Within this issue of the journal, several insights on new developments are provided in the domain of digital evidence as well as biometric features, along with details on how to use them. Using deep learning to detect the mother tongue of a speaker and traces of chemicals on clothes, and the recognition of the model of a camera from an image taken using it are some developments. The quality assurance and the validation of the results remains an important issue as new algorithms are developed.

Zeno Geradts *Netherlands Forensic Institute, Den Haag, The Netherlands University of Amsterdam, Institute of Informatics, Amsterdam, The Netherlands*
geradts@uva.nl

